# Analysis of replication factories in human cells by super-resolution light microscopy

**DOI:** 10.1186/1471-2121-10-88

**Published:** 2009-12-16

**Authors:** Zoltan Cseresnyes, Ulf Schwarz, Catherine M Green

**Affiliations:** 1 Department of Zoology, University of Cambridge, Downing Street, Cambridge, CB2 3EJ, UK; 2 Leica Microsystems, CMS GmbH, Am Friedensplatz 3, 68165 Mannheim, Germany

## Abstract

**Background:**

DNA replication in human cells is performed in discrete sub-nuclear locations known as replication foci or factories. These factories form in the nucleus during S phase and are sites of DNA synthesis and high local concentrations of enzymes required for chromatin replication. Why these structures are required, and how they are organised internally has yet to be identified. It has been difficult to analyse the structure of these factories as they are small in size and thus below the resolution limit of the standard confocal microscope. We have used stimulated emission depletion (STED) microscopy, which improves on the resolving power of the confocal microscope, to probe the structure of these factories at sub-diffraction limit resolution.

**Results:**

Using immunofluorescent imaging of PCNA (proliferating cell nuclear antigen) and RPA (replication protein A) we show that factories are smaller in size (approximately 150 nm diameter), and greater in number (up to 1400 in an early S- phase nucleus), than is determined by confocal imaging. The replication inhibitor hydroxyurea caused an approximately 40% reduction in number and a 30% increase in diameter of replication factories, changes that were not clearly identified by standard confocal imaging.

**Conclusions:**

These measurements for replication factory size now approach the dimensions suggested by electron microscopy. This agreement between these two methods, that use very different sample preparation and imaging conditions, suggests that we have arrived at a true measurement for the size of these structures. The number of individual factories present in a single nucleus that we measure using this system is greater than has been previously reported. This analysis therefore suggests that each replication factory contains fewer active replication forks than previously envisaged.

## Background

The biochemical steps required to accurately replicate the genome are well understood [1]. In recent years we have also gained an understanding of how epigenetic information may be transmitted, including the copying of DNA methylation states and the histone code from parental to daughter DNA strands during replication [2,3]. All of these processes are essential if chromosomal replication is to generate a faithful copy of the parental state, crucial for genetic and epigenetic stability and prevention of carcinogenesis [4,5].

In mammalian cells chromosomal replication occurs at discrete nuclear sites, known as replication foci or replication factories [6,7]. These structures form transiently in the nucleus and are the sites of DNA synthesis and high local concentrations of replication proteins [8-15]. Although not permanent, each factory persists in one location for a significant fraction of S phase, during which time replication proteins dynamically and independently move in and out [16-20]. The observed intra-nuclear organisation of replication factory patterns changes with progression through S phase, with early/mid S phase nuclei having a large number of small and evenly distributed factories that move to the nucleolar and cellular periphery as replication progresses to later stages [10,21,22,16]. The internal organisation of these replication factories and the possible protein-protein interactions dictating and controlling their formation, persistence and disassembly remain unclear.

Standard light microscopy is limited in resolution by diffraction and thus, as described by E. Abbe in 1873, objects closer together than approximately one half the wavelength of the light used cannot be resolved [23]. Even on a confocal microscope this prevents the resolution of objects closer than ~ 200 nm [24]. Replication factories are reported to be between 100 nm and 1 *μ*m in diameter [11,25,16] and thus light microscopes are clearly limited in their capacity to probe fine structures within these foci. Electron microscopy has also been used to investigate replication factory structures at higher resolution [13,26,27], and these studies give estimates for replication factory size between 100 and 400 nm. Here, we have applied stimulated emission depletion (STED) microscopy to analyse replication factories in human fibroblasts. STED microscopy is a technical advance that effectively breaks the resolution limit of a confocal microscope by using a donut-shaped depletion beam to deplete fluorescence from all but the very centre of the excitation spot [28,24,29]. This generates an effective excitation region smaller than can be obtained by diffraction limited optics, resulting in better definition of objects closer than Abbe's theoretical resolution limit. The advantage of this technique is that it gives high resolution analysis of immunolabelled specimens using the relatively mild sample preparation conditions of the confocal microscope. This study provides a new estimate for the maximum size of a replication factory and also for the minimum number of these factories that form at any one point in an unperturbed S phase. We have also analysed the effect that inhibition of DNA replication by hydroxyurea (HU) has on the size and number of these factories.

## Results

### Increased lateral resolution in STED mode allows more precise visualisation of replication factories

In order to visualise replication factories in human cells we selected antibodies against two key components, PCNA and RPA. RPA is a heterotrimeric single stranded DNA (ssDNA)-binding protein that associates with the template strands produced at replication forks by the action of the replicative helicase. It is important for strand stability and for recruiting other replication components to the advancing replication fork [30]. PCNA is a ring-shaped sliding clamp protein which is loaded onto template DNA at replication forks, where it acts as a platform for the recruitment of multiple enzymes required for replication [31].

**Figure 1:**
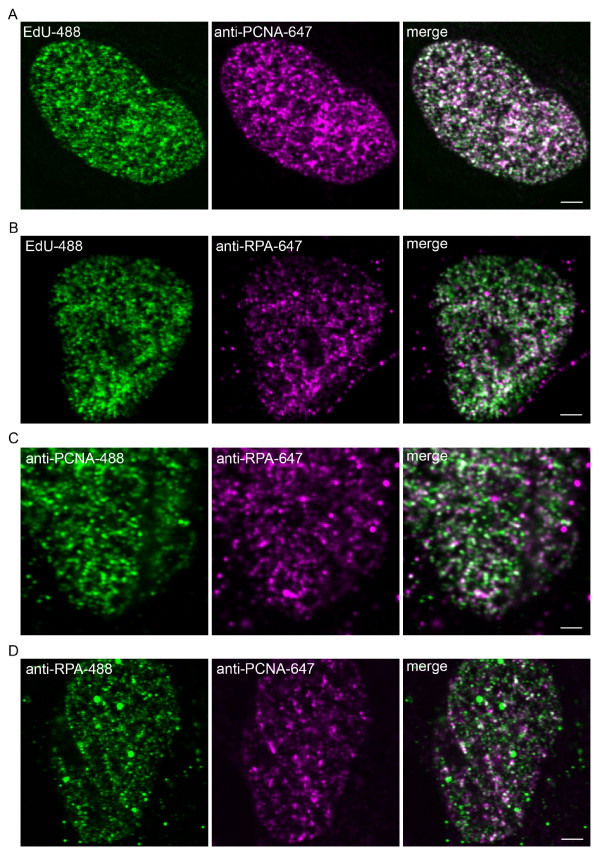
**RPA and PCNA labelled replication factories**. A and B: MRC5 cells were pulse labelled for 10 minutes with 40 *μ*M EdU to label newly synthesised DNA which was then visualised in the green channel alongside indirect immunofluorescence (in magenta) from anti-PCNA or -RPA antibodies respectively. C and D: cells were co-labelled with anti-RPA monoclonal and anti-PCNA polyclonal antibodies in green and magenta (RPA-green in C and PCNA-green in D). Scale bars = 2 *μ*m.

We first characterised the antibodies for immunofluorescent detection of replication factories using standard confocal microscopy (figure 1). MRC5 cells were pulse labelled with EdU to mark sites of DNA synthesis, then processed for immunofluorescence. EdU was detected using the Click-it cell proliferation kit (Invitrogen). Proteins were visualised indirectly using Atto 647N- or Alexa Fluor 488-linked secondary antibodies. As previously demonstrated [9,25,16], PCNA and RPA were localised in focal patterns in nuclei that were labelled with EdU and thus actively replicating DNA (figure 1A, B). Throughout this study we selected cells that showed the characteristic staining found in early S-phase, with many evenly sized nuclear replication factory distributed evenly throughout the nucleoplasm [8,10,21,22,16]. In these cells the PCNA and RPA patterns closely follow that of EdU, and the colocalisation is striking but not complete (merge panels and see supplemental figure S1 and supplemental table S1 [Additional file 1]). This is expected as PCNA is loaded at primer-template junctions, and RPA binds template DNA, whereas the EdU is incorporated as DNA is synthesised. Thus EdU labelled DNA can persist in locations after RPA or PCNA have dissociated, and RPA or PCNA can bind to regions before DNA synthesis commences. We then used the antibodies in combination to verify that the majority of these structures contained both RPA and PCNA (figure 1C and 1D). Again the colocalisation analysis shows highly similar patterns, as expected (supplemental figure S1 and table S1 [Additional file 1]). Some nuclear structures contain RPA but not PCNA. We have not excluded these from our later analyses, it is possible that they are replication factories that have recently initiated and do not yet contain active forks and PCNA, or that they are other non-replication associated RPA regions.

**Figure 2:**
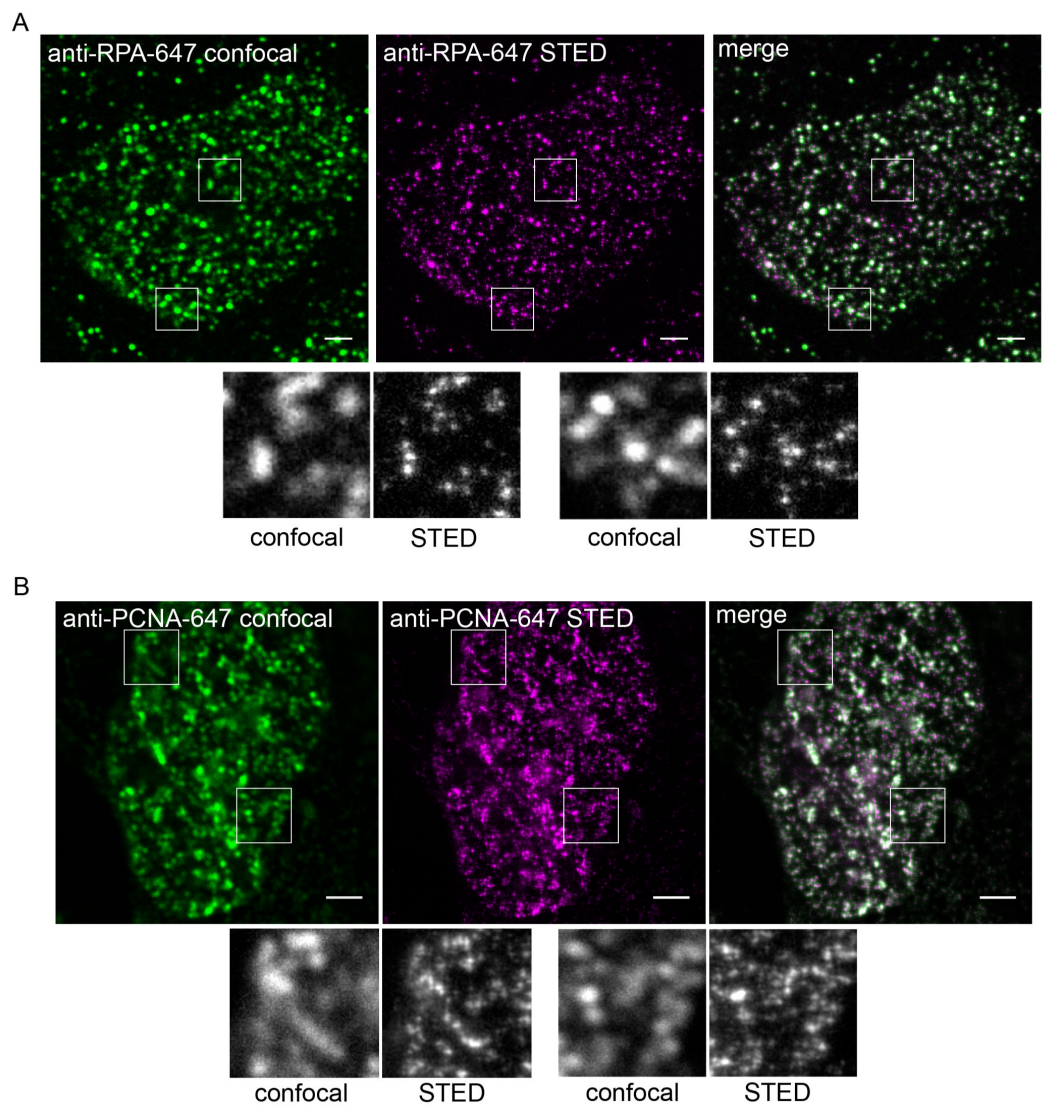
**STED imaging of replication factories**. MRC5 cells were labelled with ATTO 647-linked secondary antibodies and anti-RPA (in A) or anti-PCNA (in B) primary antibodies. Images were acquired sequentially, in normal confocal mode (green) then using the STED setup (magenta). The lower panels are magnified regions of the cells as indicated. Scale bars = 2 *μ*m.

To test whether STED microscopy can enhance the visualisation of replication structures we analysed RPA- and PCNA- labelled cells under otherwise identical imaging conditions using the confocal and STED modes on the Leica TCS STED microscope (figure 2). The STED mode utilises a depletion beam at 750 nm that effectively depletes the emission from the Atto 647N dye, resulting in a reduction of the excitation spot and consequent resolution improvement. This is a purely physical method for increasing the resolving power of the microscope - it does not depend on any mathematical processing of the images [28,24,29]. Both the RPA- (figure 2A) and PCNA- (figure 2B) labelled images were dramatically altered by the use of the STED mode. In each case the replication foci appear smaller, sharper and greater in number. This latter change results from the fact that many smaller foci can be resolved in the STED mode, which merged into one continuous structure in the confocal mode due to its limited resolution (figure 2A and 2B, lower magnified panels).

### The use of deconvolution software further improved the images obtained from the STED setup

**Figure 3:**
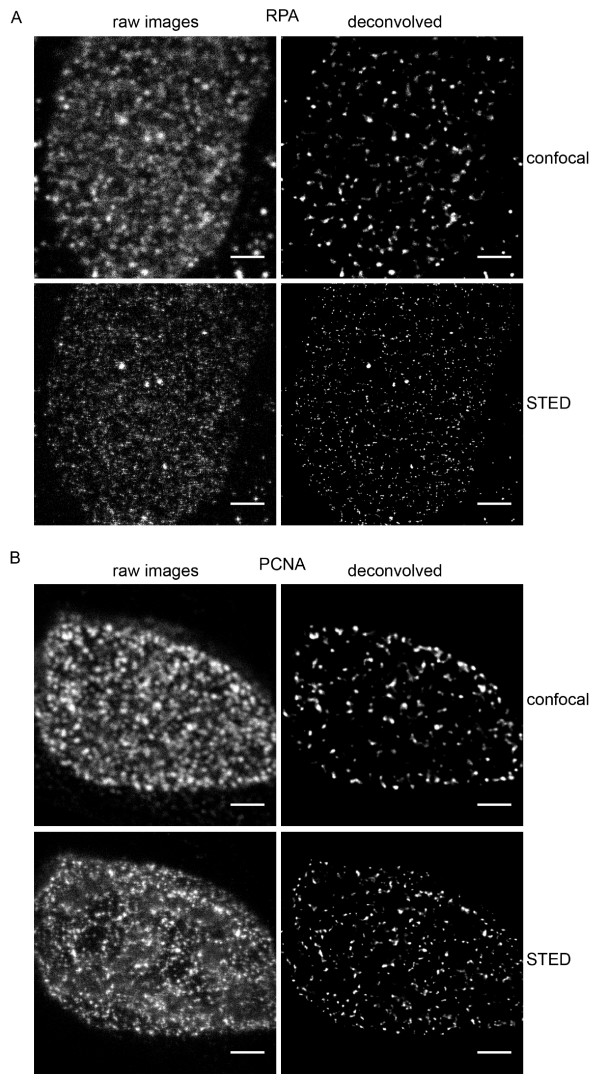
**Image restoration by deconvolution**. A series of Z slices were obtained from MRC5 cells labelled for RPA (in A) or PCNA (in B) in both confocal and STED modes. The images were then restored using the CMLE deconvolution algorithm of Huygens (SVI). Scale bars = 2 *μ*m.

Deconvolution algorithms calculate and reposition the parts of the image that are derived from degradation of the light paths due to diffraction within the instrument [32]. We used the Huygens image analysis software from Scientific Volume Imaging to apply deconvolution to the data. We used a theoretical point spread function based on microscope parameters and model ("Confocal" parameter set of Huygens) and the Classic Maximum Likelihood Estimation algorithm to restore the images. High resolution Z stacks of RPA- and PCNA- stained nuclei acquired with the confocal or STED setup were processed in identical ways (figure 3). The post acquisition processing improves the signal to noise ratio in both cases; the images have more distinct foci in each case as the processing removes background blur. As was apparent before deconvolution, the resolution improvement obtained in STED mode reduces the apparent size of the factories containing either RPA (figure 3A) or PCNA (figure 3B), and also the number of individual factories visualised is greater in STED than in confocal mode.

### STED allows a revised estimation of the number of replication factories per cell and their size

**Figure 4:**
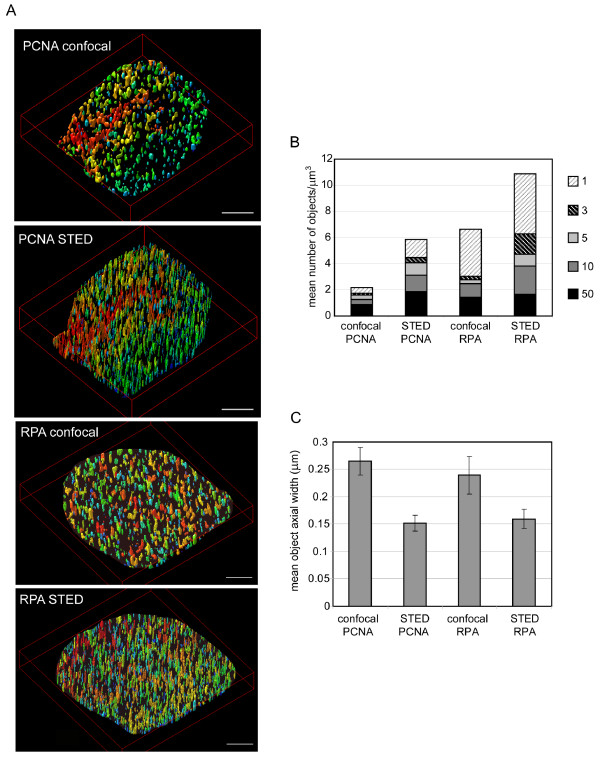
**Quantification of replication factory size and number**. Three dimensional volume renderings of the restored Z stacks were produced in SVI's Huygens imaging software (panel A - images shown are using a garbage volume of 5 voxels). Scale bars = 2 *μ*m. Colours represent increasing intensities from blue to red. The number of objects in each cell was then counted using a selection of different garbage volume thresholds. The average number of objects per *μ*m^3 ^at each garbage volume (as indicated on the right) is presented (B). The total number of objects for each threshold corresponds to the top of the appropriately shaded bar in each category. These data are also presented in table 1. The same software was used to determine the maximum axial width of an object in each category at garbage volume 5 (C). Error bars represent average deviations from the mean (n = 3-5).

Estimations for the number, and size, of replication factories in mammalian cells have previously been based on standard microscopy, confocal imaging and electron microscopy [13,15,7,16,26]. For example, using confocal imaging gives a size estimate of 250 nm for small GFP-tagged PCNA-containing replication foci [16] or 460 nm for BrdU incorporation sites [15] in mouse cells, while electron microscopy studies put sizes of replication structures below 200 nm [13,26,27,33]. Using fluorescence microscopy the number of foci present at one time in a HeLa cell was estimated at ~150-200 [13], ~250 in PtK-1 cells [10] or more recently, using confocal microscopy, at ~1000 in an early S phase mouse fibroblast [15]. Because the STED mode gives a clear improvement in the visualisation of these structures we quantified the images to give our estimate for the size and number of replication factories in cells. High resolution Z stacks (80 nm vertical step size) were acquired from early S phase nuclei stained for RPA or PCNA and the images were processed post acquisition using Huygens deconvolution (figure 4). The three dimensional nuclei were rendered and objects within a defined nuclear region of interest counted and measured using the SVI software (figure 4A). Comparisons between nuclei imaged in the confocal and STED modes are shown using different volume thresholds below which objects are ignored (called "garbage volumes" in the Huygens software). At every garbage volume the same trend is observed, the STED image has a greater number of individual objects than can be identified in the confocal mode, both for RPA and for PCNA staining (figure 4B and table 1). Table 1 presents the data analysed at a garbage volume of 5 voxels (this corresponds to ignoring objects smaller than 0.0003 *μ*m^3 ^- equivalent to 27.6 × 27.6 × 400 nm). We chose this cut-off as the Z resolution limit on this microscope is approximately 400 nm (it is not improved by STED), and thus objects smaller than this volume are well below the theoretical resolution of this microscope and are likely to be noise. The mean number of PCNA foci was increased from 1.6 to 4.1 per *μ*m^3 ^when STED mode was used instead of confocal. Similarly, for RPA there were 2.8 and 4.7 objects per *μ*m^3 ^in confocal and STED modes respectively. The number of RPA-containing factories is always greater that the number containing PCNA. This is likely due to two reasons: recently fired origins may have begun recruitment of RPA, but as this recruitment is an essential prerequisite for PCNA loading, PCNA may yet to have been recruited to levels sufficient to detect by immunofluorescence; alternatively the additional sites may represent non-replicative regions where RPA accumulates. Due to the fact that the STED system that we are using is currently limited to a single wavelength we are not able to demonstrate colocalisation between RPA and PCNA in the small replication structures that we here resolve. However, given that the signals closely correlate by confocal analysis, and our STED data shows that there are similar numbers of small RPA- and PCNA-containing structures, we think it likely that the majority of these structures contain both RPA and PCNA. Irrespective of this limitation we are able to conclude from this analysis that STED microscopy results in an updated estimation for the number of replication factories that are present in an early S phase cell, with our new estimate being approximately twice what can be derived from confocal images. In an average MRC5 nucleus (of 300 *μ*m^3^) we thus expect 1230 instead of 430 PCNA containing objects (active replication factories) and 1410 instead of 840 RPA containing objects.

**Table 1:** Replication factory numbers determined by confocal and STED microscopy.

	Confocal		STED	
	
	Mean	Range	Mean	Range
PCNA -HU	1.6	1.2 - 2.1	4.1	2.1 - 5.4

RPA -HU	2.8	1.0 - 4.1	4.7	3.0 - 7.6

PCNA +HU	1.7	1.4 - 1.8	2.6	1.5 - 3.5

RPA +HU	1.8	1.2 - 2.6	2.8	1.9 - 3.9

Cells treated with HU or untreated were analysed for RPA or PCNA containing foci by indirect immunofluorescence using confocal or STED microscopy. The images were restored using the CMLE algorithm and rendered in 3D using SVI's Huygens software, which was also used to count the objects. The average number of objects per *μ*m^3 ^(n = 3-5) and the range of values found are given for a garbage threshold volume (below which size objects are not counted) of 5 voxels (corresponding to 0.0003 *μ*m^3^).

This analysis was also used to determine the apparent size of the replication foci in each case. As above we performed this analysis using a garbage volume of 5 voxels, which resulted in clear object definition without the unwanted elimination of too many small objects. The STED images have an average replication focus size (determined as the maximum axial width of an object) of 150 nm for PCNA and 160 nm for RPA (figure 4C), an approximately 40% reduction when compared to the same cells imaged using confocal mode (object sizes 270 nm and 240 nm for PCNA and RPA). Thus STED imaging has altered our perceptions of the nature of replication factories, one factory is apparently much smaller than previously envisaged from confocal studies - indeed these sizes approach the measured size determined from electron microscopy studies [13,26].

### Hydroxyurea alters both the number and size of replication factories

**Figure 5:**
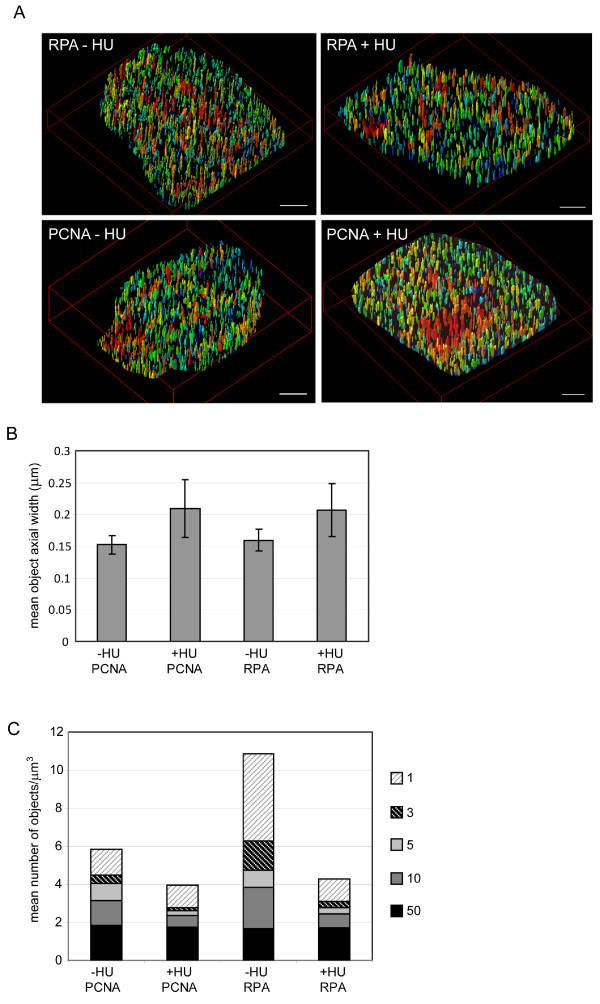
**Hydroxyurea treatment affects both replication factory number and size**. High resolution Z stacks from cells treated with hydroxyurea, or untreated, were processed as for figure 4 (A). The average maximum axial width (B) and number of objects (C) are shown, calculated exactly as in figure 3.

Finally, we applied these techniques to ask whether subtle changes in replication factories can be observed at this higher resolution. We treated cells for 12 hours with 2 mM HU, then fixed and processed them as before. 3D image stacks of cells stained for PCNA or RPA were acquired by STED, and the number and size of the factories determined as above (figure 5). We find that after HU treatment both PCNA- and RPA-containing factories are increased in size when compared to untreated cells (figure 5B). This increase in size is from 160 nm to 210 nm for RPA and from 150 nm to 210 nm for PCNA at garbage volume 5. HU also causes a decrease in the number of objects that are visible in each cell (figure 5C). At garbage volume 5 the number of objects falls from 4.7 to 2.8 per *μ*m^3 ^for RPA and from 4.1 to 2.6 per *μ*m^3 ^for PCNA. Importantly this change in the number of PCNA-containing foci, while clear in these STED images, cannot be detected if the confocal mode is used (table 1), demonstrating that the increase in resolution obtained with STED microscopy can give better insight into biological processes in vivo.

HU inhibits ribonucelotide reductase and depletes the pools of dNTPs required for DNA synthesis [34]. Initially the replicative helicase is not inhibited by this, and ssDNA is generated as the helicase unwinds DNA ahead of stalled polymerases [35]. It is therefore not unexpected that the size of the replication structures including RPA increases after HU treatment, as more RPA can be recruited to this excess ssDNA. For PCNA the increase in size is perhaps more unexpected, PCNA is loaded at primer template junctions on the leading and lagging strands, and in the absence of dNTPs no further primers will be synthesised. It may be that PCNA disengages from the primer template junction in the absence of processive synthesis and slides over the synthesised or template strands. This would cause enlargement of the PCNA bound region, either concomitant with loading of extra PCNA, or even in the absence of this. HU is unlikely to completely deplete dNTP pools and some DNA synthesis may well continue in the presence of this drug. Interestingly, both classic papers and recent studies have suggested that the replication inhibition caused by nucleotide depletion may result in the activation of origins that would otherwise remain dormant and be passively replicated [36-39]. This might be necessary in order to ensure that the genome can be completely replicated in the presence of a high rate of fork stalling events [40]. If such origin initiation events occur within a pre-existing replication factory, this could account for the increase in size of the replication foci containing both RPA and PCNA after HU treatment in these experiments. Alternatively excess ssDNA may cause the replication factories to enlarge in some other unknown manner, perhaps by altering connections between replicative polymerases and helicases and an underlying nuclear matrix scaffold. The fact that the number of observed factories decreases can be explained if the checkpoint that senses depleted nucleotide pools is more sensitive to small fluctuations in the dNTP pool than the active polymerases. In this case the initiation of new origins might be prevented while previously fired origins continue to terminate, resulting in fewer factories after HU treatment.

## Discussion

We have shown that STED microscopy can be applied to improve the imaging of small nuclear replication factories. The resolution of the STED system used in this study was approximately 75 nm (data not shown), so objects closer together than this still cannot be resolved. Nevertheless, in this example STED technology does bridge the gap between the resolution of the confocal and electron microscopes. The size that we measured here for a replication factory (~150 nm) approaches the measured diameter of replication structures visualised using electron microscopy, and for objects that truly are in the 80 - 150 nm range, STED will give an accurate picture of subcellular organisation. Of course, objects smaller than this or those that are densely clustered within the cell will still require the resolving power of the electron microscope for true visualisation. The theoretical STED resolution limit is only dependent on the power of the depletion beam and thus the photostability of the labelling dye under depletion conditions. It is therefore likely that further xy resolution improvements will be available in the relatively near future as new dyes become available [41-43]. The next steps for STED microscopy will be the development of multi-colour imaging, and resolution improvement along the Z axis. Both of these aims are likely to need significant technology development before they can be implemented on a commercial system.

In our experiments the PCNA-containing replication factories in an unperturbed early S phase have a mean size of less than 160 nm, significantly smaller than previously suggested from studies using light microscopes. We also suggest that there can be at least 1200 such factories coexisting in a normal early S phase MRC5 nucleus. Previous estimates that there are ~150 factories at any one time, and ~3000 active forks, gave rise to the supposition that one factory must contain ~20 forks [13]. A more recent study challenged these numbers [15], and our data is in support of a larger number of replication factories than was even suggested in that detailed study. If in fact the number of factories is closer to 1200 as we determine here, there would only be 2-3 forks per factory. The idea that some factories might contain so few replication forks has been previously discussed [7], and our data support this.

Is the concept of a replication factory still valid? Even with the enhanced resolution of STED, localised concentrations of replication factors are clearly visualised, so currently our answer is yes. However our data suggests that each factory in early S phase is likely to contain fewer replication forks than is generally accepted. This would mean that the amount of DNA synthesised in a coordinated fashion within a single factory may be limited to that from only one or two origins. The observed factory enlargement after hydroxyurea treatment may be due to the formation of extensive ssDNA at stalled replication forks [35], or due to the activation and recruitment into factories of otherwise dormant nearby origins [44].

There are of course many questions still to be answered before we fully understand how DNA and chromatin is accurately copied in the replication factories. Are the factories attached to structures in the nucleus? Does the DNA move? How are diverse replication events coordinated? The answering of these questions will require the formation of testable hypotheses, and the chances of developing such are increased once the description of the problem is accurate. In this report we have utilised STED microscopy to give a more precise characterisation of the nature of replication factories in human cells. This technology can also be applied to other small nuclear structures such as PML and Cajal bodies, transcription factories and focal structures formed at break sites during DNA repair. Future advances in STED, and other super-resolution microscopy technologies are likely to improve resolution still further. This will take us closer to the ultimate goal of visualising molecular machines at work in a living cell.

## Conclusions

STED microscopy dramatically improves the visualisation of replication factories under the relatively mild sample preparation conditions of the confocal microscope. This enables us to provide a firm estimate of ~150 nm for the size of RPA- and PCNA-containing replication factories in early S phase cells. This is much smaller than has been previously reported from light microscopic analysis and is very similar to the size reported from visualisation of these structures in the electron microscope. The agreement between these two very different techniques suggests that we have arrived at a correct measurement. The number of these structures is also much greater than has previously been determined (up to 1400 in a single nucleus). This suggests that there may be as few as 2 replication forks per factory. Replication inhibition by the depletion of nucleotide pools causes an increase in size and a decrease in number of replication factories.

## Methods

The cells used were MRC5 SV40 transformed human fibroblasts (a gift from A. Lehmann, University of Sussex), grown at 37 °C with 5% CO_2 _in MEM with 10% fetal calf serum (Gibco) and L-glutamine and penicillin/streptomycin (PAA). For indirect immunofluorescence cells were grown on 170 *μ*m thick glass coverslips until 50% visual confluence. Cells were washed in CSK (10 mM PIPES pH 7.0, 100 mM NaCl, 300 mM Sucrose, 3 mM MgCl_2_) then the soluble proteins were removed by incubation in CSK with 0.5% triton X-100 for 3 minutes followed by a wash in CSK and fixation in 2% freshly dissolved formaldehyde in PBS. Non-specific antibody binding sites were blocked by incubation in 5% BSA in PBS with 0.1% tween20 (blocking buffer) for 30 minutes, primary antibody incubation was for 1 hour at room temperature with antibodies (anti-RPA34 monoclonal Abcam ab16855, anti-PCNA polyclonal Abcam ab18197) diluted 1/500 in blocking buffer. After three washes secondary antibodies coupled either to Alexa Fluor 488 (Molecular Probes) or Atto 647N (Sigma) (all at 1/500 dilution) were added for 1 hour in blocking buffer. After 5 washes of 5 minutes each in PBS with 0.1% tween20 the coverslips were mounted in aquapolymount.

In some cases cells were pulse labelled for ten minutes with 40 *μ*M EdU (5-ethynyl-2-deoxyuridine) (Invitrogen), and after triton extraction and fixation as described above, sites of EdU incorporation into DNA were visualised by copper catalysed-click chemistry [45] with an amide derivative of Alexa Fluor 488 according to the manufacturer's protocol. Subsequent immunofluorescence was as above.

Where used hydroxyurea treatment was for 12 hours at 2 mM followed by immediate extraction and fixation as above.

Throughout this study we selected cells that showed the characteristic staining found in early S-phase, with many evenly sized nuclear replication factory distributed evenly throughout the nucleoplasm. All images were acquired on a Leica TCS STED equipped with an inverted microscope (DMI 6000, Leica) and a 100 × STED objective (HCX PL APO 100 × 1.4 oil STED, Leica). Confocal images were acquired using the 488 nm line for the excitation of Alexa 488 and a pulsed 635 nm laser diode (PicoQuant) to excite Atto 647N. Alexa 488 was detected using PMT 2 of the spectral detection unit with the detection range set to 495 - 550 nm and Atto 647N was detected on APD 2 equipped with a 685/40 (Semrock Bright Line, Semrock) nm bandpass filter. Imaging speed was at 400 Hz using 7 × line averaging and the pinhole was set to 0.5 Airy units. At zoom 11 and a format of 512 × 512 the resulting pixel size was 27.6 nm. Z-stacks were run at a step-size of 80 nm using the galvanometric-driven fine focussing stage of the system.

For STED microscopy all conditions were identical, but additionally the depletion laser was activated. For the stimulated emission depletion of Atto 647N the pulsed Ti:Sa IR laser (Mai Tai HP, Spectra Physics) was tuned to 750 nm and the AOM set to 100%. We calculated the xy resolution limit of our STED system to be 75 nm by imaging 20 nm crimson beads and measuring the full width half maximum of the obtained images using Leica's LAS AF quantification tool.

Confocal and STED image stacks were deconvolved using SVI's Huygens Professional package. We used the CMLE (Classic Maximum Likelihood Estimation) method of this software. The CMLE method was applied using SVI's "Confocal" optical parameters set, whereas the sampling intervals were set manually to the actual experimental values (27.6 nm for X and Y pixel sizes, 80 nm for Z step size), together with the refractive indices (1.51 for oil immersion objective and medium) and excitation/emission wavelengths. The complete parameter set was saved as a template and applied for each data set. We also performed deconvolution using a calculated PSF for the STED system (provided by Leica Microsystems). The data analysed in this way did not vary significantly from the deconvolution presented here.

Object counting was performed on the 3-D image stacks using Huygens Object Analyzer. Each data set was analysed with garbage volumes set at 1, 3, 5, 10 and 50. For each garbage volume, the fluorescence threshold was set to the value that resulted in the maximum number of objects identified. The seed value was always set at 0%, thus not setting a ceiling on voxel intensity values. Objects were counted within a pancake-shaped region of interest (ROI), where the top and bottom planes of the ROI were set to the highest and lowest Z positions of non-zero voxels of all identified objects, whereas the XY outline of the ROI was drawn manually in order to exclude stray objects from the analysis. The number of objects was divided by the volume of the ROI before calculating data average and scatter. The objects were also characterised by the axial width, as defined in Huygens: the largest width in an axial direction perpendicular to the length axis. Axial width values were only included for objects inside the ROI. The resulting datasets were analysed in Excel (Microsoft Office 2007): average values were calculated with the AVERAGE function, whereas the data scatter was characterised with the AVEDEV function.

Colocalisation analysis of doubly stained cells was performed after background subtraction using the "intensity correlation analysis" plugin for Image J from the MacMaster Biophotonics facility (full details and download available from http://www.macbiophotonics.ca/index.htm).

## Authors' contributions

CMG designed the study and prepared the samples, US advised on sample preparation and acquired the images, ZC performed the image analyses, CMG prepared the figures and wrote the manuscript, all authors edited and finalised the submission.

## Supplementary Material

Additional file 1 **Supplemental figures and tables**. Figure S1 and Table S1. *Statistical analysis of colocalisation*. Click here for file
