# Supplementary material for: Analysis of replication factories in human cells by super-resolution light microscopy
Source: BMC Cell Biol. 2009 Dec 16;10:88. doi: 10.1186/1471-2121-10-88 (PMC2803164; doi:10.1186/1471-2121-10-88)
Supplement: Additional file 1 — Supplemental figures and tables. Figure S1 and Table S1. Statistical analysis of colocalisation. [file 1471-2121-10-88-S1.PDF]

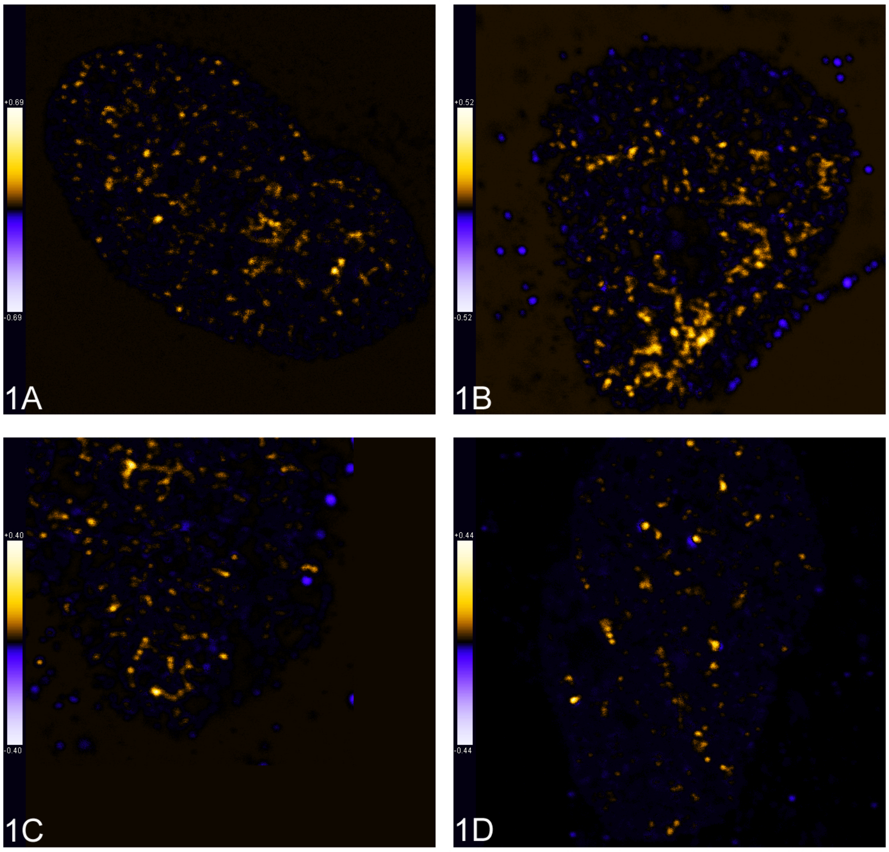

Supplemental Figure S1. Statistical analysis of colocalisation.

The pairs of confocal images (alexa-488 and atto-647) in figure 1 were compared on a pixel by pixel basis using the "intensity correlation analysis" plugin in ImageJ and the product of the differences from the mean intensity at each pixel (PDM) is plotted. The images are coloured so that positive PDM pixels (an indication of co-variance) are orange and negative PDM pixels (an indication of inverse correlation) are blue.

	1A<br>EdU-488<br>PCNA-647	1B<br>Edu-488<br>RPA-647	1C<br>PCNA-488<br>RPA-647	1D<br>RPA-488<br>PCNA-647
Pearson's Correlation coefficient (*Rr*)	0.775	0.699	0.562	0.404
Overlap coefficient (*R*)	0.883	0.843	0.774	0.564
Red pixels: green pixels	1.02	1.24	0.962	0.759
Intensity Correlation Quotient	0.321	0.295	0.275	0.25

Supplemental Table S1. Statistical analysis of colocalisation.

The pairs of confocal images (alexa-488 and atto-647) in figure 1 were compared using the "intensity correlation analysis" plugin in ImageJ. Standard measures of image colocalisation are presented for each pair.
